# The role of military service in preventing depression in China: evidence from a nationally representative longitudinal survey

**DOI:** 10.1186/s12889-023-17317-9

**Published:** 2023-12-07

**Authors:** Haoran Li, Ning Zhang, Jingya Zhang, Tao Xie, Rongxin He, Yufei Jiang, Ying Mao, Bin Zhu

**Affiliations:** 1 School of Public Policy and Administration, Xi’an Jiaotong University, Xi’an, 710049 Shaanxi China; 2 Vanke School of Public Health, Tsinghua University, Beijing, China; 3 Department of Pediatrics, Shenzhen Children’s Hospital of China Medical University, Shenzhen, China; 4 School of Public Health and Emergency Management, Southern University of Science and Technology, Shenzhen, Guangdong China

**Keywords:** Depression, Veterans, CESD, Propensity score matching, Longitudinal survey

## Abstract

**Background:**

Despite recognition in the West that military veterans experience more mental health issues than the general population, little research has focused on this subject in China. This study examined the associations between male veterans’ military experience and depression in China.

**Methods:**

A sample of 12,914 men including 669 veterans was included in the final analysis and propensity score matching, multivariable regressions and fixed effect model were used.

**Results:**

The military experience was associated with a lower likelihood of depression in male veterans. In the subgroup analysis, military experience was associated with a lower likelihood of depression among married and urban male veterans. Military experience was also associated with a lower likelihood of depression in both “junior college and above” and “below junior college” groups. In contrast, evidence was lacking regarding the associations between military experience and depression for unmarried and rural veterans.

**Conclusions:**

Individual characteristics could influence the relationship between military experience and depression in male veterans, and the mental health of veterans should be paid more attention and guaranteed.

**Supplementary Information:**

The online version contains supplementary material available at 10.1186/s12889-023-17317-9.

## Background

Veterans are vulnerable to mental health issues [1, 2]. According to existing research, veterans often exhibit higher morbidity of mental diseases than civilians, including depression and anxiety. Moreover, they are also more likely to commit suicide than the average person [3]. In the UK, veterans presented significantly higher rates of common mental disorders than the general population (23% vs. 16%); specifically, they had a 15–31% chance of being diagnosed with PTSD compared to 2–3% among the general population [1, 4–6]. Other studies have also found that depressive disorders constitute the most common mental health issues among veterans in the UK [7]. In the US, the incidence rates of mental health issues among adults were 20%, 18.6%, and 18.6% in 2010, 2012, and 2013, respectively, compared with 30.8%, 32.1%, and 32.6% among veterans who used the Veterans Health Administration [8]. Meanwhile, 10.5% and 5.6% of male veterans and non-veterans, respectively, had sought psychological intervention in the past year [9]. Worse, suicide rates among US veterans skyrocketed between 2005 and 2017, rising by 43% among men and 61% among women [10]. Similarly, using the Depression Anxiety Stress Scales (DASS), a study found that the scores of depression, anxiety, and stress among veterans in Australia were 16.09, 12.61, and 19.69, respectively, which were significantly higher than those in the general population (6.14, 4.80, and 10.29, respectively) [11]. Evidence from Korea also demonstrated more frequent depressive symptoms and rising suicidal ideation among veterans [12].

This raises the question: “Why is there such a gap in mental health between veterans and non-veterans?” According to the life course theory, the critical events in each person’s life course (e.g., entering school, getting married, joining the army, first job, etc.) comprehensively affect their subsequent health status [13, 14]. Joining the army not only changes a person’s life path and future development but also has a collateral impact on their physical and mental health, which means that it can certainly be described as a key event affecting an individual’s life course [10, 15].

Meanwhile, there is increasing interest in determining the risk factors that can contribute to the mental health issues of veterans because it is increasingly being accepted that risk factors play a critical role in the development of mental health issues [16]. Notably, being female, having low educational attainment, having drinking and smoking habits, or not owning a suitable house is proven to be associated with a higher risk of depression among veterans [10, 17–19]. Conversely, social support, particularly from spouses, is considered a potential protective factor for veterans [20]. Moreover, certain aspects of military culture, such as its emphasis on resilience, self-reliance, and avoidance of expressing vulnerable feelings, can cause mental health issues among veterans and treatment avoidance, sometimes leading to suicide [21, 22].

However, Chinese veterans carry an elite status. In the context of the continuous crisis of authority in modern times, the social status of soldiers in China has risen, and they have gradually become the protagonists of its history. Likewise, since the founding of People’s Republic of China (PRC), the social status of servicemembers has improved. There is no denying that they have transformed into a group respected by society. In reality, joining the army is a turning point in one’s life, and could make one’s family or neighbors proud [23]. The Chinese government has also provided comprehensive security for veterans. In 2018, the Ministry of Veterans Affairs, PRC, was established to strengthen the security system for veterans [24]. The Law of the People’s Republic of China on the Protection of Ex-Servicemen came into force on January 1, 2021, providing security for ex-servicemembers in terms of placement, education and training, employment and entrepreneurship, and pension and preferential treatment.

As an important event in the life course, military experience has not received much scholarly attention, and there are few studies on the mental health status of Chinese soldiers after they leave the army [25]. To fill this research gap, this study examined male Chinese veterans from 2012‒2018, adopting propensity score matching (PSM) to explore the relationship between military experience and depression among male veterans in China. Specifically, we aimed to answer the following questions: What is the relationship between military experience and depression in male veterans in China? What factors contribute to the relationship between military experience and male veterans’ depression; Is there heterogeneity in the association between military experience and male veterans?

## Methods

### Study participants

**Fig. 1:**
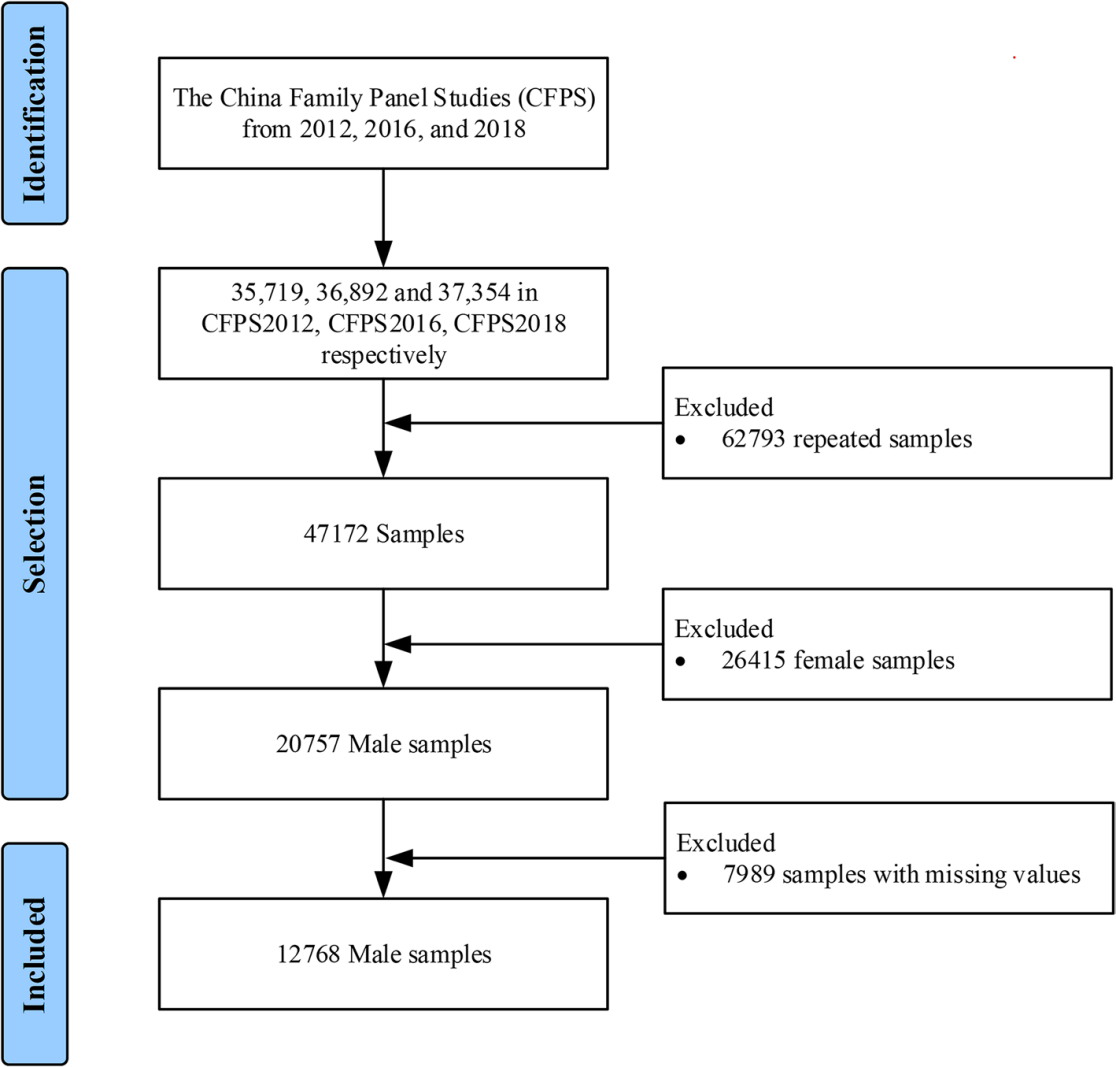
Flow chart of the study participants. Data from the China Family Panel Studies (CFPS) from 2012, 2016, and 2018 were applied. Notes: Due to the scarcity of female veterans, only male individuals were included

According to data released by the National Bureau of Statistics of China on April 27, 2012, the proportion of male and female soldiers in active service of the People’s Liberation Army is 95.74% and 4.26%. As there were few female veterans in the data source, this study focused on male veterans in China. Data from the China Family Panel Studies (CFPS) from 2012, 2016, and 2018 were used in this study. CFPS2014 was not included because it did not use the Center for Epidemiologic Studies Depression Scale (CESD). The CFPS, a nationally representative sample covering China’s 25 provinces/regions and 95% of the population, is implemented by the Chinese Center for Social Science Surveys (ISSS) at Peking University [26]. In the three rounds of research in 2012, 2016, and 2018, the sample sizes were 35,719, 36,892, and 37,354, respectively. Because CFPS conducted a follow-up survey on certain participants, there were repeated participants in the databases of 2012, 2016, and 2018. For the same participants included in two or three waves, we only retained relevant data in the latest year. After combining the three datasets, we removed all data on female respondents, resulting in a total of 20,757 participants. Subsequently, we removed participants with missing values or data that were not applicable and identified 12,768 participants as the final study participants. Figure 1 presents the detailed selection process.

### Measures

#### Depression

This study measured the mental health of veterans by ascertaining whether they experienced depression. In 2012, 2016, and 2018, the CFPS used the CESD to measure depression among adults, including 20 questions about the self-rated mental health of respondents. Radloff proposed a depressive rating scale ranging from 0 to 60 [27]. A score higher than 28 indicates severe depressive symptoms [27]. Accordingly, adults who scored higher than 28 on the CESD scale of CFPS2012 and 2016 were categorized as “depressed”. Therefore, we used the dummy variable (whether depression) as the main dependent variable of this paper. Simultaneously, we also reported regression results using individual depression scores as the dependent variable in the manuscript and Supplementary materials.

#### Military experience

Although there are questions about the time of joining the army, the CPFS questionnaire has several missing values. However, in its 2012, 2016, and 2018 questionnaires, the CFPS questionnaire also asks, “Are you a veteran or not?” Therefore, our sample comprising those who answered “yes” were defined as “having military experience.”

#### Covariates

Referring to previous studies [28, 29], covariables in this association included age, household register (rural or urban), home address (East, Middle, or West China, according to *the National Development and Reform Commission*), employment (employed or unemployed), marriage (married or unmarried), educational attainment, smoking (whether one smoked in the past month), drinking (whether one drank three times a week or more in the past month), and family size (the logarithm of the total number of people in a household). Meanwhile, we divided the participants into subgroups A (married and unmarried), B (junior college and above and below junior college), and C (urban and rural) according to marital status, education level, and household registration.

### Statistical analysis

For Model 1, we used logistic regression to analyze the impact of military experience on depression without controlling the covariates. Model 2 extended Model 1 by including all the covariates described in subsection 2.2.3 (Covariates). Model 3 used unbalanced fixed effects panel data model to test long-term relationships and improve the robustness of the results [30]. Models 4, 5 and 6 were analyzed based on the results of PSM. Similar to Models 1, 2 and 3, Model 4 tested the impact of military experience on the depression experienced by male veterans without covariates, and Model 5 and 6 included all the covariates described in subsection 2.2.3 (Covariates). We used the same analysis in subgroups. With PSM and covariates, Model 5 and 6 was the main model we focused on.

To enhance the credibility of the results, we used PSM to screen the participants. PSM is a common method used in observational research to effectively reduce selection bias [31]. The 1:2 nearest-neighbor matching ratio with replacement was used to match depressed veterans with normal veterans. If the standardized difference was less than 10%, it was considered an indicator of balance [32]. Following PSM, we deleted the participants that failed to match. After the matching, we also adopted logistic regression to examine the effect of military experience on depression among male veterans, accounting for the pairing between veteran and non-veteran groups.

In the sensitivity analysis, 1:2 nearest-neighbor matching with replacement and a caliper of 0.0001 and 1:1 nearest-neighbor matching was used to determine the robustness of the results. We also verified the robustness of the results by including deployment duration as a covariate.

All statistical analyses were performed using Stata 15.1 (Stata Corp., LLC). A *p*-value of < 0.05 was considered statistically significant.

## Results

### Study sample

This study included 47,214 participants from CFPS 2012, 2016, and 2018; 12,914 participants remained after excluding the participants with missing information or unqualified conditions. The final sample for the regression analyses following PSM amounted to 1,684. Figure 1 presents the details of the selection.

### Propensity score matching

Using PSM, we matched the veteran group, which included 669 male veterans, with a non-veteran group of 1015 men without military experience. Table 1 and Table S4 shows descriptive statistics.

**Table 1:** The descriptive statistics and T-test result of depression and individual depression score between the veteran and non-veteran groups after propensity score matching in different subgroups

**Dependent variable**	**Subgroups**		**Matched veterans**			**Matched non-veterans**		**T-test results between groups**			
		**N**	**Mean /%**	**95% CI**	**N**	**Mean / %**	**95% CI**	**Mean / %**	**SE**	**95% CI**	***P*-value**
**Depression**	**All participants**	669	62.63%	58.96–66.31%	984	69.85%	45.91–67.02%	7.19%	2.35%	2.57–11.80%	0.0023
	**Unmarried**	81	81.48%	72.84–90.12%	142	81.69%	75.25–88.13%	0.21%	5.42%	-10.47–10.89%	0.9693
	**Married**	588	60.03%	56.06–64.00%	880	68.41%	65.33–71.49%	8.38%	2.53%	3.41–13.34%	0.0010
	**Junior college and above**	69	59.42%	47.54–71.30%	104	75.00%	66.54–83.46%	15.58%	7.14%	1.49–29.67%	0.0304
	**Below junior college**	600	63.00%	59.13–66.87%	690	71.34%	68.37–75.11%	8.74%	2.60%	3.64–13.84%	0.0008
	**Rural**	270	68.52%	62.94–74.09%	440	71.81%	67.60–76.04%	3.30%	3.53%	-3.62–10.22%	0.3497
	**Urban**	399	58.65%	53.79–63.50%	570	67.54%	46.86–63.69%	8.90%	3.13%	2.76–15.03%	0.0045
**Individual depression score**	**All**	669	31.12	30.53–31.70	984	32.51	32.01–33.01	1.39	0.40	0.61–2.17	0.0005
	**Unmarried**	81	35.38	33.31–37.45	142	34.89	33.37–36.41	-0.50	1.28	-3.02–2.03	0.6998
	**Married**	588	30.53	29.94–31.12	880	32.10	31.60–32.61	1.58	0.10	0.79–2.36	0.0001
	**Junior college and above**	69	29.77	28.21–31.32	104	31.94	30.66–33.23	2.17	1.02	0.17–4.18	0.0341
	**Below junior college**	600	31.27	7.86–30.64	690	32.76	32.16–33.36	1.49	0.44	0.62–2.35	0.0008
	**Rural**	270	32.37	31.38–33.36	440	33.30	32.52–34.07	0.93	0.64	-0.33–2.19	0.1485
	**Urban**	399	30.27	31.39–32.69	570	32.04	31.39–32.69	1.77	0.49	0.81–2.74	0.0003

1) SE, Standard errors

2) The results of 1:2 nearest-neighbor matching with replacement was used

**Fig. 2:**
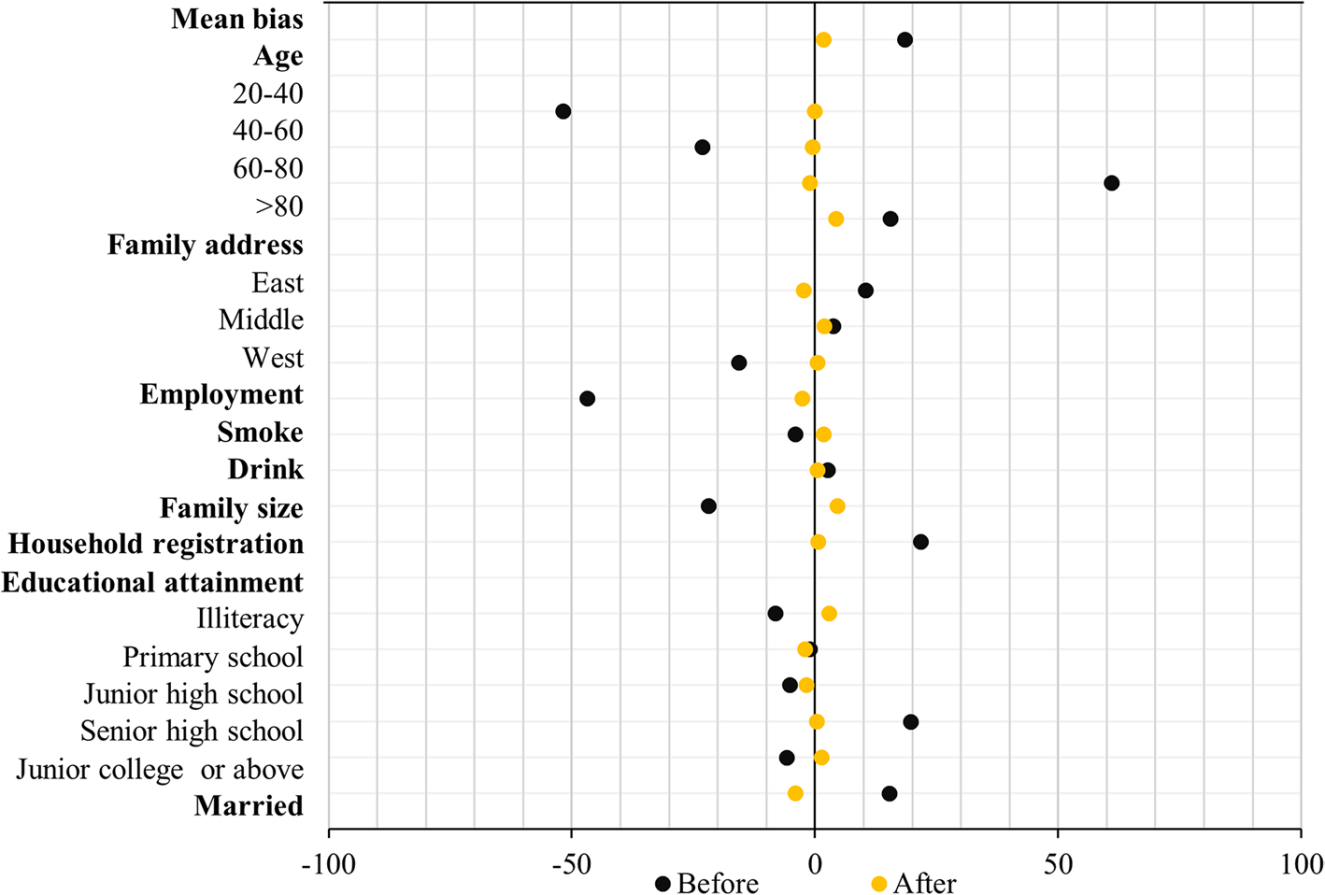
Standardized differences between the veteran and non-veteran groups before and after the 1:2 nearest-neighbor matching with replacement. Notes: 1) The black dots and yellow dots represent standardized differences before and after matching respectively. 2) Details are provided in Supplementary Table S1

Figure 2 presents the standardized differences between the veteran and non-veteran groups before and after 1:2 nearest-neighbor matching (details are provided in Supplementary Table S1). After matching, the standardized differences of all covariates were significantly decreased. By reducing the standard differences, PSM allowed comparisons between veteran and non-veteran groups.

### Association estimation in all the participants

Table 1 shows the descriptive statistics and T-test result of depression and individual depression score in the veteran and non-veteran groups.

Table 2 reports the results of regression analysis of military experience and depression before and after 1:2 nearest-neighbor PSM. Covariates are also included in the regression and reported. In Model 5, military experience was associated with a lower likelihood of depression among male veterans (OR = 0.73, 95%CI = 0.59 ~ 0.90, *p* < 0.01). In Model 6, military experience was also associated with a lower likelihood of depression among male veterans (Coef. = -0.07, 95%CI = -0.11 ~ -0.02, *p* < 0.01). This conclusion still held true in Models 1‒4. The results indicate that age may be a potential risk factor. In Model 5 and 6, the 20–40 years old age group (OR = 2.73, 95%CI = 1.37 ~ 5.44, *p* < 0.01; Coef. = 0.20, 95%CI = 0.05 ~ 0.35, *p* < 0.01), and the 40–60 years old age group were associated with a high likelihood of depression (OR = 2.00, 95%CI = 1.08 ~ 3.70, p < 0.05; Coef. = 0.14, 95%CI = 0.01 ~ 0.28, *p* < 0.05). Living in cities can also become a risk factor for depression (OR = 0.66, 95%CI = 0.52 ~ 0.83, *p* < 0.001; Coef. = -0.09, 95%CI = -0.14 ~ 0.04, *p* < 0.001). However, being married was negatively associated with the likelihood of depression (OR = 0.55, 95%CI = 0.37 ~ 0.82, *p* < 0.01; Coef. = -0.11, 95%CI = -0.19 ~ -0.04, *p* < 0.01). The results of the sensitivity analysis are reported in Supplementary Tables S2 and S3.

**Table 2:** The association between the military experience and depression in male veterans before and after propensity score matching

Characteristic	Before matching (n = 12,768)						After matching (*n* = 1684)					
	**Model 1: crude**		**Model 2: logistic**		**Model 3: fixed effect**		**Model 4: crude**		**Model 5: logistic**		**Model 6: fixed effect**	
	**OR**	**95%CI**	**OR/Coef**	**95%CI**	**Coef**	**95%CI**	**OR**	**95%CI**	**OR/Coef**	**95%CI**	**Coef**	**95%CI**
**Military experience**	0.65***	0.55 ~ 0.77	0.79**	0.67 ~ 0.94	-0.05**	-0.09 ~ -0.02	0.72**	0.59 ~ 0.89	0.73**	0.59 ~ 0.90	-0.07**	-0.11 ~ -0.02
**Age**												
20–40			2.02***	1.48 ~ 2.74	0.14***	0.07 ~ 0.20			2.73**	1.37 ~ 5.44	0.20**	0.05 ~ 0.35
40–60			1.55**	1.15 ~ 2.09	0.09**	0.03 ~ 0.15			2.00*	1.08 ~ 3.70	0.14*	0.01 ~ 0.28
60–80			1.26	0.94 ~ 1.68	0.05	-0.01 ~ 0.11			1.39	0.80 ~ 2.42	0.07	-0.05 ~ 0.20
**Household registration**			0.89***	0.78 ~ 0.92	-0.03***	-0.05 ~ -0.02			0.66***	0.52 ~ 0.83	-0.09***	-0.14 ~ -0.04
**Home address**												
East			0.59***	0.53 ~ 0.65	-0.10***	-0.12 ~ -0.08			0.74*	0.56 ~ 0.96	-0.07*	-0.12 ~ -0.01
Middle			0.71***	0.64 ~ 0.79	-0.06***	-0.08 ~ -0.04			0.91	0.68 ~ 1.23	-0.02	-0.08 ~ 0.04
**Employment**			1.01	0.89 ~ 1.13	0.001	-0.02 ~ 0.03			0.88	0.68 ~ 1.15	-0.03	-0.08 ~ 0.03
**Smoke**			1.03	0.95 ~ 1.12	0.01	-0.01 ~ 0.02			1.09	0.88 ~ 1.35	0.02	-0.03 ~ 0.06
**Drinking**			0.93	0.85 ~ 1.01	-0.02	-0.03 ~ 0.00			0.85	0.67 ~ 1.06	-0.03	-0.08 ~ 0.02
**Family size**			0.98	0.96 ~ 1.00	-0.004*	-0.01 ~ 0.00			0.99	0.94 ~ 1.05	0.00	-0.01 ~ 0.01
**Educational attainment**												
Illiteracy			1.41***	1.19 ~ 1.67	0.07***	0.03 ~ 0.10			1.46	0.91 ~ 2.34	0.08	-0.02 ~ 0.18
Primary school			1.20*	1.03 ~ 1.40	0.04*	0.00 ~ 0.07			1.21	0.79 ~ 1.85	0.04	-0.05 ~ 0.13
Junior high school			1.11	0.96 ~ 1.27	0.02	-0.01 ~ 0.05			0.92	0.63 ~ 1.36	-0.02	-0.10 ~ 0.07
Senior high school			0.95	0.82 ~ 1.11	-0.01	-0.04 ~ 0.02			0.81	0.55 ~ 1.21	-0.05	-0.13 ~ 0.04
**Marriage**			0.63***	0.56 ~ 0.71	-0.08***	-0.10 ~ -0.06			0.55**	0.37 ~ 0.82	-0.11**	-0.19 ~ -0.04

1) *P < 0.05, **P < 0.01, ***P < 0.001. *Coef.* coefficient, *OR* odds ratio

2) The results of 1:2 nearest-neighbor matching with replacement was used

3) Model 1 computed odds ratios without any statistical adjustment in unmatched participants. Model 2 computed odds ratios adjusting for covariates using multivariate logistic regressions in unmatched participants. Model 3 computed coefficient adjusting for covariates using fixed effect model in unmatched participants. Model 4 computed odds ratios without any statistical adjustments in matched participants. Model 5 computed odds ratios adjusting for covariates in multivariate logistic regressions in matched participants. Model 6 computed coefficient adjusting for covariates using fixed effect model in matched participants

Table 3 reports the results of regression analysis of military experience and individual depression score before and after 1:2 nearest-neighbor PSM. Covariates are also included in the regression and reported. In Model 5, military experience was associated with lower depression score among male veterans (Coef. = -1.05, 95%CI = -1.78 ~ -0.32, *p* < 0.01). In Model 6, military experience was also associated with a lower likelihood of depression among male veterans (Coef. = -1.07, 95%CI = -1.80 ~ -0.34, *p* < 0.01). This conclusion still held true in Models 1‒4.

**Table 3:** The association between the military experience and individual depression scores in male veterans before and after propensity score matching

Characteristic	Before matching (*n* = 12,768)						After matching (*n* = 1684)					
	**Model 1: crude**		**Model 2: OLS**		**Model 3: fixed effect**		**Model 4: crude**		**Model 5: OLS**		**Model 6: fixed effect**	
	**Coef**	**95%CI**	**Coef**	**95%CI**	**Coef**	**95%CI**	**Coef**	**95%CI**	**Coef**	**95%CI**	**Coef**	**95%CI**
**Military experience**	-1.41***	-2.01 ~ -0.81	-0.90**	-1.50 ~ -0.31	-0.91**	-1.51 ~ -0.31	-1.09**	-1.85 ~ -0.34	-1.05**	-1.78 ~ -0.32	-1.07*	-1.80 ~ -0.34
**Age**												
20–40			2.18***	1.11 ~ 3.25	2.18***	1.11 ~ 3.25			2.65*	0.28 ~ 5.02	2.66*	0.28 ~ 5.05
40–60			1.91***	0.86 ~ 2.95	1.90***	0.86 ~ 2.95			2.53*	0.38 ~ 4.69	2.53*	0.37 ~ 4.68
60–80			1.22*	0.20 ~ 2.23	1.22**	0.21 ~ 2.24			1.43	-0.52 ~ 3.39	1.47	-0.49 ~ 3.42
**Household registration**			-0.91***	-1.19 ~ -0.63	-0.91***	-1.19 ~ -0.63			-1.68***	-2.47 ~ -0.90	-1.72***	-2.50 ~ -0.93
**Home address**												
East			-1.85***	-2.17 ~ -1.52	-1.86***	-2.18 ~ -1.53			-1.60**	-2.51 ~ -0.68	-1.58**	-2.50 ~ -0.67
Middle			-1.17***	-1.52 ~ -0.82	-1.17***	-1.53 ~ -0.82			-0.85	-1.84 ~ 0.14	-0.84	-1.84 ~ 0.15
**Employment**			-1.00***	-1.40 ~ -0.61	-1.02***	-1.42 ~ -0.62			-1.09*	-1.99 ~ -0.18	-1.11*	-2.02 ~ -0.20
**Smoke**			0.43**	0.16 ~ 0.70	0.43**	0.16 ~ 0.70			0.49	-0.25 ~ 1.22	0.48	-0.26 ~ 1.22
**Drinking**			-0.57***	-0.86 ~ -0.28	-0.57***	-0.86 ~ -0.27			-0.58	-1.37 ~ 0.22	-0.59	-1.39 ~ 0.21
**Family size**			-0.12**	-0.19 ~ -0.05	-0.12***	-0.19 ~ -0.05			-0.05	-0.24 ~ 0.15	-0.05	-0.24 ~ 0.15
**Educational attainment**												
Illiteracy			2.54***	1.98 ~ 3.11	2.53***	1.97 ~ 3.09			3.53***	1.92 ~ 5.14	3.50***	1.89 ~ 5.12
Primary school			1.38***	0.87 ~ 1.89	1.37***	0.86 ~ 1.88			1.28	-0.18 ~ 2.73	1.27	-0.19 ~ 2.73
Junior high school			0.97***	0.50 ~ 1.44	0.97***	0.50 ~ 1.43			0.79	-0.55 ~ 2.14	0.76	-0.58 ~ 2.11
Senior high school			0.17	-0.34 ~ 0.68	0.16	-0.35 ~ 0.67			0.13	-1.22 ~ 1.49	0.09	-1.27 ~ 1.45
**Marriage**			-2.53***	-2.91 ~ -2.15	-2.52***	-2.90 ~ -2.14			-3.11***	-4.34 ~ -1.89	-3.07***	-4.30 ~ -1.84

1) *P < 0.05, **P < 0.01, ***P < 0.001. *Coef.* coefficient

2) The results of 1:2 nearest-neighbor matching with replacement was used

3) Model 1 computed coefficient without any statistical adjustment in unmatched participants. Model 2 computed coefficient adjusting for covariates using multivariate logistic regressions in unmatched participants. Model 3 computed coefficient adjusting for covariates using fixed effect model in unmatched participants. Model 4 computed coefficient without any statistical adjustments in matched participants. Model 5 computed coefficient adjusting for covariates in multivariate logistic regressions in matched participants. Model 6 computed coefficient adjusting for covariates using fixed effect model in matched participants

### Subgroup analysis

#### Propensity score matching

With PSM, we matched male veterans with men without military experience in the non-veteran group groups in different subgroups. Supplementary Table S4 shows the descriptive statistics. Supplementary Figure S1 presents the standardized differences between the veteran and non-veteran groups before and after 1:2 nearest-neighbor matching with replacement (details are provided in Supplementary Table S5).

### Association estimation in subgroups

**Fig. 3:**
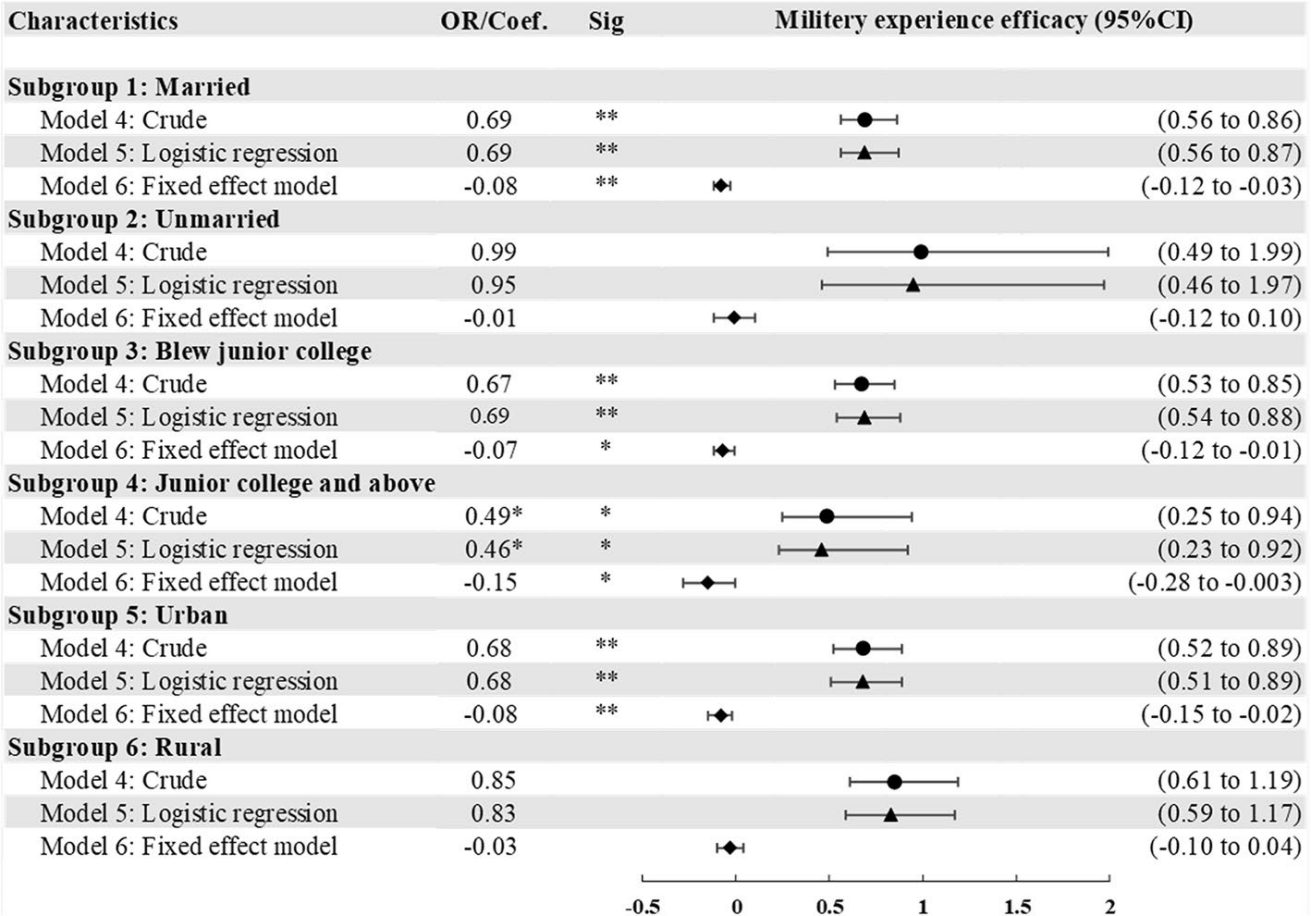
The association between military experience and depression in different subgroups after propensity score matching. Notes: 1) *P < 0.05, **P < 0.01, ***P < 0.001. Coef., coefficient; OR, odds ratio. 2) The results of 1:2 nearest-neighbor matching with replacement was used. 3) Details are provided in Supplementary Table S6, S7 and S8. 4) Model 4 computed odds ratios without any statistical adjustments in matched participants. Model 5 computed odds ratios adjusting for covariates in multivariate logistic regressions in matched participants. Model 6 computed coefficient adjusting for covariates using fixed effect model in matched participants

Figure 3 demonstrated the after-matching results of subgroups by marriage, educational attainment, and urban/rural residence in Models 4, 5 and 6 (details of the regression results before matching are provided in Supplementary Tables S6, S7 and S8).

For married male veterans, military experience was associated with a lower likelihood of depression in Model 5 and 6 (OR = 0.69, 95%CI = 0.56 ~ 0.87, *p* < 0.01; Coef. = -0.08, 95%CI = -0.12 ~ -0.03, *p* < 0.01). However, the association between military experience of unmarried male veterans and depression was not significant. Military experience was associated with a lower likelihood of depression in both junior college and above (OR = 0.46, 95%CI = 0.23 ~ 0.92, *p* < 0.05; Coef. = -0.15, 95%CI = -0.29 ~ -0.003, *p* < 0.05) and below junior college groups (OR = 0.69, 95%CI = 0.54 ~ 0.88, *p* < 0.01; Coef. = -0.07, 95%CI = -0.12 ~ -0.01, *p* < 0.05) in Model 5 and 6. Compared with the insignificant association between depression and the military experience of male veterans who lived in rural areas, military experience was linked with a lower likelihood of depression among male veterans who lived in urban areas (OR = 0.68, 95%CI = 0.51 ~ 0.89, *p* < 0.01; Coef. = -0.08, 95%CI = -0.15 ~ -0.02, *p* < 0.01) in Model 5 and 6. These results are valid in different matching methods.

**Fig. 4:**
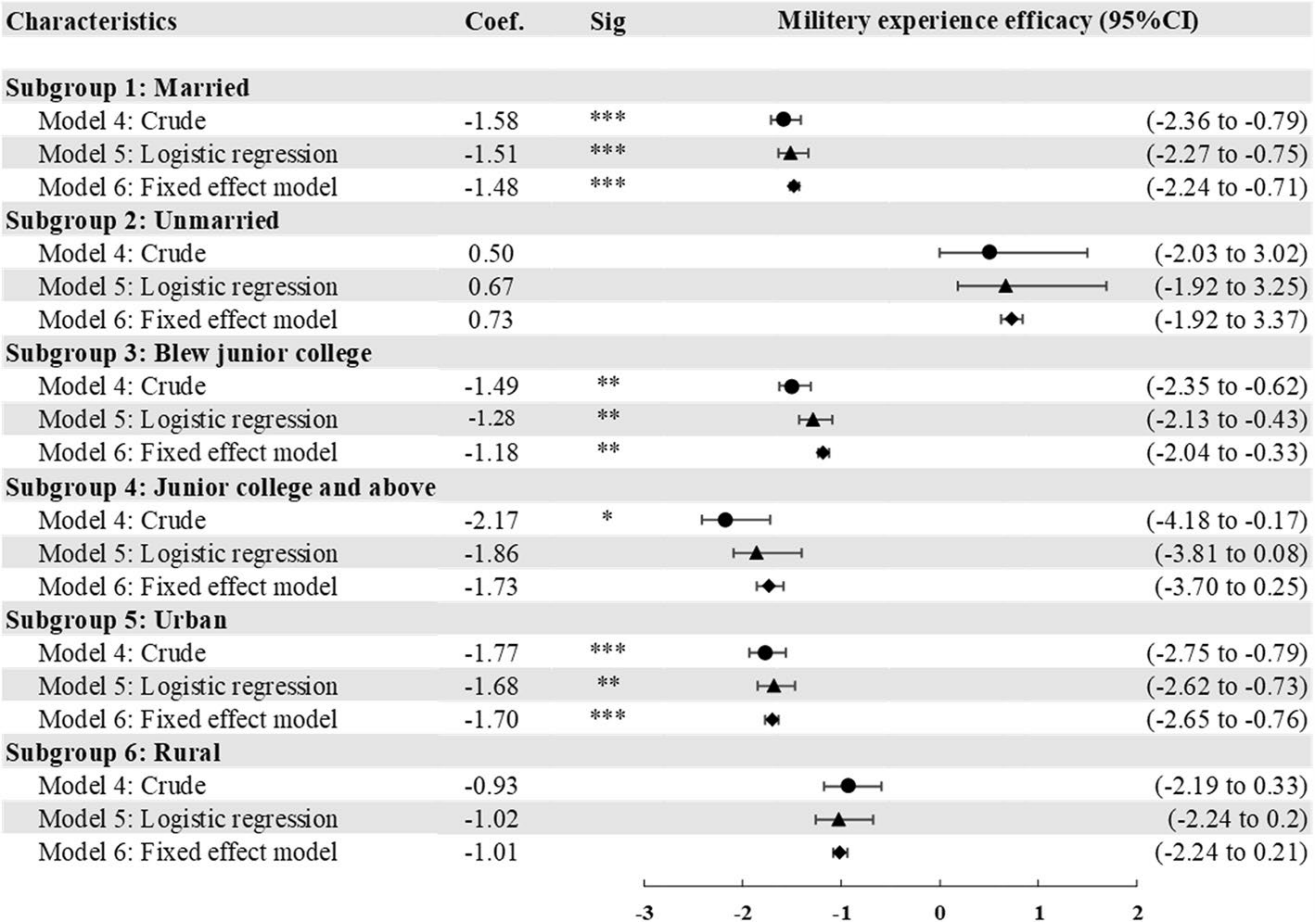
The association between military experience and individual depression score in different subgroups after propensity score matching. Notes: 1) *P < 0.05, **P < 0.01, ***P < 0.001. Coef., coefficient. 2) The results of 1:2 nearest-neighbor matching with replacement was used. 3) Details are provided in Supplementary Table S9, S10 and S11. 4) Model 4 computed coefficient without any statistical adjustments in matched participants. Model 5 computed coefficient adjusting for covariates in multivariate logistic regressions in matched participants. Model 6 computed coefficient adjusting for covariates using fixed effect model in matched participants

Changing the dependent variable to individual depression score, Fig. 4 demonstrated the after-matching results of subgroups by marriage, educational attainment, and urban/rural residence in Models 4, 5 and 6 (details of the regression results before matching are provided in Supplementary Tables S9, S10 and S11). The relationship between military experience and lower individual depression score was significant among married, below junior college, and urban subgroups.

## Discussion

Mental health issues of veterans have been studied in many countries. However, we have little information about how military experience affects depression among male veterans in China. To fill this research gap, we used data from the 2012–2018 CFPS to estimate the association between military experience and depression among Chinese male veterans.

First, evidence from our study indicated that the military experience of Chinese male veterans has a positive impact on the incidence of depression, which is partially different from other countries [2, 3]. We believe that distinct elements of military culture, such as education, alcohol, tobacco, and tolerance, may be of key importance, because these forms of military culture may affect the individuals’ lives in the long-term [33]. Compared with the British army, Chinese soldiers are better educated, and the Chinese government began recruiting volunteers at universities in 2013 [33, 34]. The US military considers tobacco use a right, and alcohol and cannabis use were prevalent among their recruits [19, 35]. Conversely, the *Internal Affairs Regulations of the Chinese People’s Liberation Army* require that there be no excessive drinking among soldiers and advocate quitting smoking. Moreover, military culture in other countries, which promote characteristics such as toughness, self-reliance, or other traditional male gender norms, may lead to stigma and discrimination when facing mental health issues among the military [36, 37]. This kind of military culture renders mental health issues harder to recognize than medical problems that are physical in nature.

Moreover, admitting to having mental health issues harmed the careers of soldiers who returned from the US mission in Bosnia, which may have caused them to avoid treatment-seeking [21, 38]. In contrast, China’s military culture is more tolerant of soldiers’ mental health issues. According to the *Internal Affairs Regulations of the Chinese People's Liberation Army*, platoon leaders’ responsibilities include monitoring the psychological status of platoon members. Moreover, mental health services at all levels are required to be strengthened to improve the psychological quality of officers and soldiers. Such an inclusive environment may make enable veterans to seek help for their mental health issues, which may ease their depression.

In addition, social culture itself constitutes a set of unwritten rules that constrains our behavior and influences our life, contributing to the difference [39]. Under the pressure of being invaded, the status of Chinese soldiers advanced by leaps and bounds due to their contribution to the victory of the wars, and gradually established their elite status after the founding of New China. This status was reflected in income, advantages in the marriage market, and interpersonal relationships, etc. [15, 23, 28]. The advantages these veterans have established in society may decrease their depression.

However, the soldiers of other countries do not appear to gain much from their military experience in terms of either income or marriage market [40, 41]. Studies have even demonstrated that military experience might reduce the future earnings of veterans [42]. Moreover, since 2014, the media tend to shape veterans as health victims in the US, which may have created public stereotypes about veterans as “bad” or “mad” [43]. Such stereotypes may be a barrier to veterans pursuing higher goals because civilian students and institutions perceive them as “broken,” “dispirited,” or “crazy and violent” [39, 44]. This negative image of veterans can lead to social stereotypes that might prevent veterans from receiving the respect they expect by virtue of being veterans, increasing their susceptibility to depression.

Additionally, conflicts such as the Korean, Vietnam, and Iraq wars may increase veterans’ depression and other mental health issues [12, 45, 46]. Evidence from US, UK, Canada and Australia also demonstrate the relationship between deployment and mental health issues [6, 47–49]. After the Sino-Vietnamese war in the early 1980s, China had not participated in a war for more than 40 years; in contrast, the US is currently involved in multiple wars [50]. Staying away from deployment during military service helps veterans avoid combat-related stress and pain, which may be a potential reason for the negative correlation between military experience and depression among Chinese veterans.

Heterogeneities in the association by marriage, educational attainment, and urban/rural residence were also revealed in this study. Married and urban male veterans are less likely to be diagnosed with depression. Male veterans with all educational attainments exhibited a lower risk of depression, but the difference was more significant in the below junior college group, which may be because people with fewer educational qualifications were more likely to be transformed and promoted by military experience. Moreover, there were no associations between military experience and depression for unmarried and rural veterans.

Like previous studies, our study demonstrated that being married is a protective factor against mental health issues among veterans [20] because strong social support is a predictor of positive mental health [51]. In conclusion, those with strong social support, particularly by their spouse, may be more receptive to stress, which could protect them against mental health issues [33, 52]. Meanwhile, being married may represent a sense of responsibility to others developed in the military, which could potentially protect veterans against depression [53].

Simultaneously, because of China’s household registration system, its urban population has significant advantages in employment types, income, benefits from social welfare policies, government support, and health resources compared with the rural population [54–56]. Therefore, the pursuit of a better living environment, and the desire to pursue urban household registration and change their economic and social fates may drive many rural people to join the military [57]. However, once they transition from rural to urban registration, it is hard to revert owing to the Chinese household registration policy. The participants in our study with rural household registration did not obtain urban registration through military experience. Thus, when they found that military experience did not change their circumstances and that they had to stay in the countryside, the impact of military experience on their depression was minimal. For those born in cities rich in social resources, the desire to practice skills and improve their overall abilities may motivate them to join the army [58]. While military service did enhance their skills and abilities, it had a significant effect on reducing their likelihood of depression [59].

### Limitations

Despite several strengths, this study has some limitations. First, depression was diagnosed by CES-D, a self-reported scale, which could cause information bias [60]. Second, it is difficult to obtain large and corresponding data related to veterans. Large standard errors may result from the small number of unmarried men studied, which might have affected the significance of the results [61]. In addition, the CFPS does not permit us to describe Chinese male veterans in a more comprehensive manner. Our findings would be strengthened if more covariates, such as economic capability and stable housing, could be included in our study [62, 63]. Moreover, PSM with a range of covariates was used to balance the measured confounding factors; however, we could not control for bias from unobserved confounding factors, which is a limitation common to almost all previous epidemiological studies [64]. Third, to increase the sample volume, we removed duplicate participants from CFPS2012 to 2018; therefore, this study is cross-sectional. However, the possibility that regressions of participants from different years may have caused deviations in results owing to unobservable temporal effects. Thus, we believe a cohort study might be more appropriate to strengthen our findings. Further, we were unable to involve female veterans in our study because female soldiers constitute a small portion of the Chinese military.

## Conclusion

Using nationally representative longitudinal data, our study indicates that the military experience of Chinese male veterans is associated with a lower likelihood of depression. After grouping the participants by marriage, educational attainment, and urban/rural residence, we found evidence suggesting that military experience is linked to a lower likelihood of depression among married, urban, junior college and above, and below junior college groups. However, no significant association was found between military experience and depression among unmarried and rural male veterans. Future research should involve female participants, assess subdivided military experiences, and explore the mechanism by which military experience helps suppress depression.

### Supplementary Information

**Additional file 1.**

## Data Availability

The original data presented in the study are publicly available. This data can be found here: [http://www.isss.pku.edu.cn/cfps/].
