# Supplementary material for: The role of military service in preventing depression in China: evidence from a nationally representative longitudinal survey
Source: BMC Public Health. 2023 Dec 7;23:2447. doi: 10.1186/s12889-023-17317-9 (PMC10702066; doi:10.1186/s12889-023-17317-9)
Supplement: Supplementary file 1 — Additional file 1. [file 12889_2023_17317_MOESM1_ESM.docx]

**Online Supplemental Materials**

**Technical Note S1**. Computation of Standardized Differences in Covariates

For a dichotomous variable such as tobacco user, the standardized difference is defined as:

$$d=\frac{(\hat{p}_{treated}-\hat{p}_{control} )}{\sqrt{\frac{\hat{p}_{treated}\left( 1-\hat{p}_{treated} \right)+\hat{p}_{control}(1-\hat{p}_{control})}{2}}}$$

Where $\hat{p}_{treated}$ and $\hat{p}_{control}$ denote the percentage of the dichotomous variable taking the value of 1 among infants exposed and unexposed to prenatal cannabis use disorder, respectively.

**Table S1.** Standardized differences in covariates of all participants. Standardized differences (in percentage points).

| **Characteristics** | **Before matching** | | **After matching** | |
| --- | --- | --- | --- | --- |
|  | SD (%) | P-value | SD (%) | P-value |
| **Mean bias** | 18.5 | <0.001 | 1.8 | 0.999 |
| **Age** |  |  |  |  |
| 20-40 | -51.8 | <0.001 | <0.1 | 1.000 |
| 40-60 | -23.1 | <0.001 | -0.5 | 0.929 |
| 60-80 | 61.0 | <0.001 | -1.1 | 0.848 |
| Above 80 | 15.5 | <0.001 | 4.4 | 0.484 |
| **Household registration** | 21.7 | <0.001 | 0.6 | 0.911 |
| **Home address** |  |  |  |  |
| East | 10.5 | 0.008 | -2.3 | 0.682 |
| Middle | 3.8 | 0.339 | 2.0 | 0.718 |
| West | -15.6 | <0.001 | 0.5 | 0.923 |
| **Employment** | -46.8 | <0.001 | -2.6 | 0.669 |
| **Smoke** | -4.0 | 0.316 | 1.8 | 0.742 |
| **Drinking** | 2.7 | 0.490 | 0.5 | 0.929 |
| **Family size** | -21.9 | <0.001 | 4.6 | 0.368 |
| **Educational attainment** |  |  |  |  |
| Illiteracy | -8.2 | 0.045 | 2.9 | 0.578 |
| Primary school | -1.1 | 0.787 | -2.0 | 0.720 |
| Junior high school | -5.2 | 0.193 | -1.8 | 0.744 |
| Senior high school | 19.6 | <0.001 | 0.4 | 0.948 |
| Junior college and above | -5.9 | 0.151 | 1.4 | 0.786 |
| **Marriage** | 15.3 | -4.0 | <0.001 | 0.414 |

Notes:

1) Veteran group included all veterans. Non-veteran group included all ordinary individuals.

**Table S2.** The results of regression after different matching methods of all participants.

| **Characteristic** | **After 1:2 & caliper=0.0001 matching (n=1640)** | | | | | | **After 1:1 matching (n=1185)** | | | | | |
| --- | --- | --- | --- | --- | --- | --- | --- | --- | --- | --- | --- | --- |
|  | **Model 4: Crude** | | **Model 5: Logistic** | | **Model 6: Fixed effect** | | **Model 4: Crude** | | **Model 5: Logistic** | | **Model 6: Fixed effect** | |
|  | **OR** | **95%CI** | **OR/Coef.** | **95%CI** | **OR/Coef.** | **95%CI** | **OR** | **95%CI** | **OR/Coef.** | **95%CI** | **OR/Coef.** | **95%CI** |
| **Military experience** | 0.71** | 0.58~0.88 | 0.72** | 0.58~0.89 | -0.07** | -0.11~-0.02 | 0.71** | 0.56~0.91 | 0.72* | 0.56~0.93 | -0.06* | -0.12~-0.01 |
| **Age** |  |  |  |  |  |  |  |  |  |  |  |  |
| 20-40 |  |  | 3.23** | 1.57~6.67 | 0.24** | 0.09~0.40 |  |  | 2.66* | 1.18~6.00 | 0.18* | 0.004~0.35 |
| 40-60 |  |  | 2.35* | 1.23~4.50 | 0.18* | 0.04~0.32 |  |  | 1.69 | 0.83~3.46 | 0.10 | -0.06~0.25 |
| 60-80 |  |  | 1.60 | 0.88~2.89 | 0.11 | -0.02~0.24 |  |  | 1.06 | 0.56~2.00 | 0.01 | -0.13~0.15 |
| **Household registration** |  |  | 0.72* | 0.55~0.95 | -0.09*** | -0.14~-0.05 |  |  | 0.73 | 0.53~1.01 | -0.09** | -0.15~-0.04 |
| **Home address** |  |  |  |  |  |  |  |  |  |  |  |  |
| East |  |  | 0.92 | 0.68~1.25 | -0.07* | -0.13~-0.01 |  |  | 0.93 | 0.65~1.33 | -0.07 | -0.13~0.00 |
| Middle |  |  | 0.89 | 0.68~1.16 | -0.02 | -0.08~0.05 |  |  | 0.85 | 0.62~1.17 | -0.01 | -0.09~0.06 |
| **Employment** |  |  | 1.04 | 0.87~1.30 | -0.03 | -0.09~0.03 |  |  | 1.06 | 0.82~1.37 | -0.04 | -0.11~0.03 |
| **Smoke** |  |  | 0.84 | 0.67~1.06 | 0.01 | -0.04~0.06 |  |  | 0.79 | 0.60~1.04 | 0.01 | -0.04~0.07 |
| **Drinking** |  |  | 0.99 | 0.94~1.05 | -0.03 | -0.09~0.02 |  |  | 1.00 | 0.94~1.07 | -0.05 | -0.11~0.01 |
| **Family size** |  |  | 0.65*** | 0.52~0.82 | 0.00 | -0.01~0.01 |  |  | 0.66** | 0.50~0.87 | 0.00 | -0.01~0.02 |
| **Educational attainment** |  |  |  |  |  |  |  |  |  |  |  |  |
| Illiteracy |  |  | 1.47 | 0.91~2.39 | 0.08 | -0.02~0.18 |  |  | 1.28 | 0.74~2.21 | 0.05 | -0.07~0.17 |
| Primary school |  |  | 1.24 | 0.80~1.91 | 0.05 | -0.05~0.14 |  |  | 1.37 | 0.83~2.26 | 0.07 | -0.04~0.18 |
| Junior high school |  |  | 0.91 | 0.61~1.36 | -0.02 | -0.11~0.07 |  |  | 0.95 | 0.60~1.50 | -0.02 | -0.11~0.08 |
| Senior high school |  |  | 0.81 | 0.54~1.21 | -0.05 | -0.13~0.04 |  |  | 0.79 | 0.50~1.25 | -0.05 | -0.15~0.05 |
| **Marriage** |  |  | 0.57** | 0.38~0.86 | -0.11** | -0.19~-0.03 |  |  | 0.58* | 0.36~0.93 | -0.11* | -0.20~-0.01 |

Notes:

1) *P<0.05, **P<0.01, ***P<0.001. Coef., coefficient; OR, odds ratio.

2) The results of 1:2 & caliper=0.0001 matching and 1:1 matching was used.

3) Model 4 computed odds ratios without any statistical adjustments in matched participants. Model 5 computed odds ratios adjusting for covariates in multivariate logistic regressions in matched participants. Model 6 computed coefficient adjusting for covariates (including deployment duration) using fixed effect model in matched participants.

**Table S3** the regression results after bringing deployment duration into the regression.

| **Characteristic** | **Regression with employment duration (n=12764)** | | | | | |
| --- | --- | --- | --- | --- | --- | --- |
|  | **Model 1: crude** | | **Model 2: logistic** | | **Model 3: fixed effect** | |
|  | **OR** | **95%CI** | **OR/Coef.** | **95%CI** | **OR/Coef.** | **95%CI** |
| **Military experience** | 0.65*** | 0.56~0.77 | 0.71** | 0.55~0.92 | -0.08** | -0.13~-0.02 |
| **Deployment duration** |  |  | 1.02 | 0.98~1.06 | 0.004 | -0.004~0.01 |
| **Age** |  |  |  |  |  |  |
| 20-40 |  |  | 2.06*** | 1.52~2.81 | 0.14*** | 0.08~0.21 |
| 40-60 |  |  | 1.58** | 1.17~2.13 | 0.09** | 0.03~0.15 |
| 60-80 |  |  | 1.28 | 0.96~1.72 | 0.05 | -0.01~0.11 |
| **Household registration** |  |  | 0.85*** | 0.78~0.92 | -0.03*** | -0.05~-0.02 |
| **Home address** |  |  |  |  |  |  |
| East |  |  | 0.59*** | 0.53~0.65 | -0.10*** | -0.12~-0.08 |
| Middle |  |  | 0.71*** | 0.64~0.79 | -0.06*** | -0.08~-0.04 |
| **Employment** |  |  | 1.01 | 0.90~1.13 | 0.002 | -0.02~0.03 |
| **Smoke** |  |  | 1.03 | 0.95~1.12 | 0.01 | -0.01~0.02 |
| **Drinking** |  |  | 0.93 | 0.85~1.01 | -0.02 | -0.03~0.002 |
| **Family size** |  |  | 0.98 | 0.96~1.00 | -0.004* | -0.01~0.00 |
| **Educational attainment** |  |  |  |  |  |  |
| Illiteracy |  |  | 1.42*** | 1.19~1.68 | 0.07*** | 0.03~0.10 |
| Primary school |  |  | 1.21* | 1.04~1.41 | 0.04* | 0.01~0.07 |
| Junior high school |  |  | 1.11 | 0.97~1.28 | 0.02 | -0.01~0.05 |
| Senior high school |  |  | 0.96 | 0.83~1.12 | -0.01 | -0.04~0.02 |
| **Marriage** |  |  | 0.63*** | 0.56~0.72 | -0.08*** | -0.10~-0.06 |

Notes:

1) *P<0.05, **P<0.01, ***P<0.001. Coef., coefficient; OR, odds ratio.

2) Model 1 computed odds ratios without any statistical adjustment. Model 2 computed odds ratios adjusting for covariates (including deployment duration) using multivariate logistic regressions. Model 3 computed coefficient adjusting for covariates (including deployment duration) using fixed effect model.

**Table S4.** Descriptive statistics in different subgroups.

| **Characteristic** | **Before matching**  **(12768)** | | **After matching** | | | | | | | | | | | | | | |
| --- | --- | --- | --- | --- | --- | --- | --- | --- | --- | --- | --- | --- | --- | --- | --- | --- | --- |
|  |  |  | **ALL**  **(N=1684)** | | **Unmarried**  **(N=223)** | | **Married**  **(N=1468)** | | **Junior college and above**  **(N=173)** | | **Below Junior college**  **(N=1290)** | | **Rural**  **(N=710)** | | **Urban**  **(N=969)** | | |
|  | **Mean or %** | **Standard difference** | **Mean or %** | **Standard difference** | **Mean or %** | **Standard difference** | **Mean or %** | **Standard difference** | **Mean or %** | **Standard difference** | **Mean or %** | **Standard difference** | **Mean or %** | **Standard difference** | **Mean or %** | **Standard difference** |  |
| **Military experience** | 5.24% | 0.22 | 39.73% | 0.49 | 36.32% | 0.48 | 40.01% | 0.49 | 39.88% | 0.49 | 46.51% | 0.50 | 38.03% | 0.49 | 41.18% | 0.49 |  |
| **Age** |  |  |  |  |  |  |  |  |  |  |  |  |  |  |  |  |  |
| 20-40 | 29.47% | 0.46 | 11.58% | 0.32 | 43.95% | 0.50 | 6.20% | 0.24 | 24.28% | 0.43 | 10.93% | 0.31 | 8.31% | 0.28 | 13.52% | 0.34 |  |
| 40-60 | 41.25% | 0.49 | 30.76% | 0.46 | 13.90% | 0.35 | 33.58% | 0.47 | 42.77% | 0.50 | 30.62% | 0.46 | 29.86% | 0.46 | 31.89% | 0.47 |  |
| 60-80 | 27.45% | 0.45 | 53.74% | 0.50 | 31.39% | 0.47 | 57.63% | 0.49 | 27.75% | 0.45 | 54.57% | 0.50 | 59.44% | 0.49 | 49.85% | 0.50 |  |
| Above 80 | 1.82% | 0.13 | 3.92% | 0.19 | 10.76% | 0.31 | 2.59% | 0.16 | 5.20% | 0.22 | 3.88% | 0.19 | 2.39% | 0.15 | 4.74% | 0.21 |  |
| **Household registration** |  |  |  |  |  |  |  |  |  |  |  |  |  |  |  |  |  |
| **Home address** | 42.14% | 0.49 | 46.32% | 0.50 | 45.29% | 0.50 | 46.73% | 0.50 | 47.98% | 0.50 | 43.72% | 0.50 | 44.51% | 0.50 | 50.05% | 0.50 |  |
| East | 27.68% | 0.45 | 28.86% | 0.45 | 26.01% | 0.44 | 29.22% | 0.45 | 31.21% | 0.46 | 29.84% | 0.46 | 29.15% | 0.45 | 27.55% | 0.45 |  |
| Middle | 30.17% | 0.46 | 24.82% | 0.43 | 28.70% | 0.45 | 24.05% | 0.43 | 20.81% | 0.41 | 26.43% | 0.44 | 26.34% | 0.44 | 22.39% | 0.42 |  |
| West | 82.82% | 0.38 | 65.38% | 0.48 | 63.67% | 0.48 | 65.53% | 0.48 | 66.47% | 0.47 | 66.12% | 0.47 | 77.89% | 0.42 | 57.59% | 0.49 |  |
| **Employment** | 57.17% | 0.49 | 54.28% | 0.50 | 58.30% | 0.49 | 54.29% | 0.50 | 43.93% | 49.77% | 55.74% | 0.50 | 56.76% | 0.50 | 54.08% | 0.50 |  |
| **Smoke** | 28.57% | 0.45 | 30.11% | 0.46 | 19.28% | 0.40 | 32.08% | 0.47 | 34.68% | 0.48 | 30.85% | 0.46 | 29.15% | 0.45 | 30.34% | 0.46 |  |
| **Drinking** | 4.10 | 2.08 | 3.72 | 1.93 | 2.93 | 1.91 | 3.84 | 1.90 | 3.10 | 1.36 | 3.89 | 2.08 | 3.88 | 2.03 | 3.53% | 1.75 |  |
| **Family size** | 49.44% | 0.50 | 58.55% | 0.49 | 58.74% | 0.49 | 57.63% | 0.49 | 87.28% | 0.33 | 54.88% | 0.50 |  |  |  |  |  |
| **Educational attainment** |  |  |  |  |  |  |  |  |  |  |  |  |  |  |  |  |  |
| Illiteracy | 17.03% | 0.38 | 13.95% | 0.35 | 12.56% | 0.33 | 14.10% | 0.35 |  |  |  |  | 18.87% | 0.39 | 11.25% | 0.32 |  |
| Primary school | 22.70% | 0.42 | 22.57% | 0.42 | 20.63% | 0.41 | 22.96% | 0.42 |  |  |  |  | 27.89% | 0.45 | 18.16% | 0.39 |  |
| Junior high school | 32.18% | 0.47 | 30.05% | 0.46 | 27.35% | 0.45 | 30.72% | 0.46 |  |  |  |  | 32.54% | 0.47 | 26.93% | 0.44 |  |
| Senior high school | 16.01% | 0.37 | 23.16% | 0.42 | 29.60% | 0.46 | 22.34% | 0.42 |  |  |  |  | 17.46% | 0.38 | 29.00% | 0.45 |  |
| Junior college and above | 12.08% | 0.33 | 10.27% | 0.30 | 9.87% | 0.30 | 9.88% | 0.30 |  |  |  |  | 3.24% | 0.18 | 14.65% | 0.35 |  |
| **Marriage** | 82.74% | 0.38 | 87.82% | 0.33 |  |  |  |  | 85.55% | 0.35 | 85.58% | 0.35 | 86.90% | 0.34 | 86.69% | 0.34 |  |

Note: Subgroups include unmarried group, married group, junior college and above group, below junior college group, rural group and urban group.

**Table S5.** Standardized differences in covariates of different subgroups. SD: Standardized differences (in percentage points).

| **Characteristics** | **Subgroup A** | | | | | | | | **Subgroup B** | | | | | | | | **Subgroup C** | | | | | | | | |
| --- | --- | --- | --- | --- | --- | --- | --- | --- | --- | --- | --- | --- | --- | --- | --- | --- | --- | --- | --- | --- | --- | --- | --- | --- | --- |
|  | **Unmarried** | | | | **Married** | | | | **Junior college and above** | | | | **Below junior college** | | | | **Rural** | | | | **Urban** | | | | |
|  | **Before Matching** | | **After Matching** | | **Before Matching** | | **After Matching** | | **Before Matching** | | **After Matching** | | **Before Matching** | | **After Matching** | | **Before Matching** | | **After Matching** | | **Before Matching** | | **After Matching** | |  |
|  | **SDiff (%)** | **P-value** | **SDiff (%)** | **P-value** | **SDiff (%)** | **P-value** | **SDiff (%)** | **P-value** | **SDiff (%)** | **P-value** | **SDiff (%)** | **P-value** | **SDiff (%)** | **P-value** | **SDiff (%)** | **P-value** | **SDiff (%)** | **P-value** | **SDiff (%)** | **P-value** | **SDiff (%)** | **P-value** | **SDiff (%)** | **P-value** |  |
| **Mean bias** | 16.6 | <0.001 | 4.1 | 1.000 | 19.8 | <0.001 | 2.5 | 0.995 | 37.7 | <0.001 | 3.7 | 0.999 | 22.4 | <0.001 | 2.2 | 0.989 | 20.0 | <0.001 | 2.7 | 0.999 | 16.5 | <0.001 | 4.2 | 0.920 |  |
| **Age** |  |  |  |  |  |  |  |  |  |  |  |  |  |  |  |  |  |  |  |  |  |  |  |  |  |
| 20-40 | -30.9 | 0.006 | 3.7 | 0.814 | -54.4 | <0.001 | 0.7 | 0.846 | -113.5 | <0.001 | <0.1 | 1.000 | -43.3 | <0.001 | 1.6 | 0.723 | -55.1 | <0.001 | 0.5 | 0.934 | -52.4 | <0.001 | 1.5 | 0.785 |  |
| 40-60 | -16.5 | 0.173 | -1.7 | 0.907 | -27.8 | <0.001 | -0.7 | 0.902 | 44.8 | <0.001 | -4.7 | 0.799 | -31.7 | <0.001 | -1.9 | 0.729 | -31.6 | <0.001 | -2.0 | 0.812 | -16.5 | 0.002 | -0.5 | 0.940 |  |
| 60-80 | 31.9 | 0.002 | -5.8 | 0.738 | 63.7 | <0.001 | -2.7 | 0.658 | 64.9 | <0.001 | 5.9 | 0.781 | 60.5 | <0.001 | -2.5 | 0.683 | 73.3 | <0.001 | 1.6 | 0.860 | 54.8 | <0.001 | -5.1 | 0.501 |  |
| Above 80 | 29.7 | <0.001 | 4.8 | 0.799 | 13.8 | <0.001 | 9.0 | 0.152 | 24.4 | 0.003 | <0.1 | 1.000 | 14.4 | <0.001 | 8.9 | 0.153 | 6.5 | 0.236 | <0.1 | 1.000 | 19.4 | <0.001 | 10.4 | 0.184 |  |
| **Household registration** | 20.3 | 0.075 | 2.5 | 0.874 | 22.1 | <0.001 | 1.4 | 0.813 | 28.1 | 0.041 | 2.0 | 0.891 | 23.3 | <0.001 | -0.7 | 0.907 |  |  |  |  |  |  |  |  |  |
| **Home address** |  |  |  |  |  |  |  |  |  |  |  |  |  |  |  |  |  |  |  |  |  |  |  |  |  |
| East | 5.6 | 0.622 | 1.2 | 0.938 | 11.3 | 0.007 | -5.1 | 0.382 | -8.1 | 0.510 | -7.2 | 0.673 | 13.1 | 0.002 | -0.3 | 0.954 | 20.5 | 0.001 | -8.0 | 0.365 | -1.9 | 0.709 | -6.8 | 0.340 |  |
| Middle | 6.0 | 0.591 | <0.1 | 1.000 | 3.0 | 0.482 | 3.7 | 0.520 | 12.1 | 0.315 | 4.7 | 0.787 | 2.8 | 0.502 | -0.4 | 0.949 | 6.6 | 0.281 | 3.3 | 0.705 | 1.1 | 0.832 | 5.8 | 0.407 |  |
| West | -11.8 | 0.309 | -1.3 | 0.930 | -15.8 | <0.001 | 1.9 | 0.728 | -3.7 | 0.768 | 3.5 | 0.832 | -17.3 | <0.001 | 0.7 | 0.892 | -27.6 | <0.001 | 5.2 | 0.522 | 1.1 | 0.824 | 1.8 | 0.795 |  |
| **Employment** | -38.6 | <0.001 | -6.9 | 0.684 | -48.8 | <0.001 | -2.2 | 0.738 | -63.3 | <0.001 | 1.8 | 0.930 | -44.7 | <0.001 | -3.3 | 0.609 | -32.8 | <0.001 | -3.9 | 0.683 | -51.7 | <0.001 | -6.1 | 0.432 |  |
| **Smoke** | 6.6 | 0.559 | -1.2 | 0.937 | -5.8 | 0.169 | -1.2 | 0.838 | 6.1 | 0.617 | <0.1 | 1.000 | -5.9 | 0.158 | 1.0 | 0.862 | -5.8 | 0.349 | 1.1 | 0.896 | -0.6 | 0.915 | -3.5 | 0.619 |  |
| **Drinking** | 1.0 | 0.930 | 10.6 | 0.485 | 1.6 | 0.697 | 0.6 | 0.925 | 42.3 | <0.001 | 3.4 | 0.859 | -2.2 | 0.609 | -1.3 | 0.825 | 4.7 | 0.447 | 5.7 | 0.510 | 1.5 | 0.776 | -2.5 | 0.728 |  |
| **Family size** | 0.4 | 0.965 | 14.3 | 0.324 | -29.4 | <0.001 | 4.7 | 0.390 | -25.5 | 0.066 | 13.6 | 0.320 | -22.2 | <0.001 | 3.3 | 0.550 | -20.6 | 0.001 | 7.6 | 0.353 | -19.1 | <0.001 | 7.9 | 0.213 |  |
| **Educational attainment** |  |  |  |  |  |  |  |  |  |  |  |  |  |  |  |  |  |  |  |  |  |  |  |  |  |
| Illiteracy | -9.0 | 0.444 | 3.4 | 0.816 | -8.2 | 0.062 | 1.6 | 0.769 |  |  |  |  |  |  |  |  | -11.0 | 0.089 | -0.5 | 0.956 | 0.4 | 0.942 | 1.6 | 0.821 |  |
| Primary school | 2.3 | 0.835 | -8.0 | 0.626 | -2.4 | 0.573 | -1.6 | 0.782 |  |  |  |  |  |  |  |  | 4.9 | 0.430 | <0.1 | 1.000 | -1.3 | 0.802 | -5.6 | 0.438 |  |
| Junior high school | 6.2 | 0.578 | 1.4 | 0.930 | -7.9 | 0.065 | -1.8 | 0.752 |  |  |  |  |  |  |  |  | 1.6 | 0.800 | 2.4 | 0.784 | -9.7 | 0.065 | 1.6 | 0.812 |  |
| Senior high school | 29.9 | 0.004 | <0.1 | 1.000 | 18.6 | <0.001 | 0.2 | 0.972 |  |  |  |  |  |  |  |  | 10.7 | 0.069 | -1.1 | 0.907 | 21.6 | <0.001 | -2.7 | 0.726 |  |
| Junior college and above | -36.0 | 0.005 | 3.4 | 0.788 | 1.7 | 0.687 | 2.8 | 0.627 |  |  |  |  |  |  |  |  | -11.7 | 0.093 | -2.9 | 0.700 | -11.4 | 0.034 | 5.3 | 0.422 |  |
| **Marriage** |  |  |  |  |  |  |  |  | 53.5 | <0.001 | 1.8 | 0.897 | 9.3 | 0.033 | -2.9 | 0.590 | 14.7 | 0.026 | 0.5 | 0.947 | 16.2 | 0.003 | -2.5 | 0.702 |  |

Notes:

1) Veteran group included all veterans. Non-veteran group included all ordinary individuals.

**Table S6.** The association between the military experience and depression in male veterans before and after propensity score matching in unmarried and married group.

| **Analysis** | **Models** | **Unmarried** | | | **Married** | | |
| --- | --- | --- | --- | --- | --- | --- | --- |
|  |  | **OR/Coef.**  **(95%, CI)** | **P-value** | **N** | **OR/Coef.**  **(95% CI)** | **P-value** | **N** |
| **Before**  **matching** | **Model 1**  **Crude** | 1.08  (0.61~1.91) | 0.795 | 2204 | 0.64  (0.54~0.76) | <0.001 | 10564 |
|  | **Model 2**  **Logistic** | 1.19  (0.67~2.14) | 0.550 | 2204 | 0.77  (0.64~0.91) | 0.003 | 10564 |
|  | **Model 3**  **Fixed effect** | 0.02  (-0.07~0.11) | 0.621 | 2204 | -0.06  (-0.10~-0.02) | 0.002 | 10564 |
| **After**  **matching** | **Model 4**  **Crude** | 0.99  (0.49~1.99) | 0.969 | 223 | 0.69  (0.56~0.86) | 0.001 | 1468 |
|  | **Model 5**  **Logistic** | 0.95  (0.46~1.97) | 0.889 | 223 | 0.69  (0.56~0.87) | 0.001 | 1468 |
|  | **Model 6**  **Fixed effect** | -0.01  (-0.12~0.10) | 0.900 | 223 | -0.08  (-0.12~-0.03) | 0.003 | 1468 |

Notes:

1) *p<0.05, **p<0.01, ***p<0.001. Coef., coefficient; OR, odds ratio.

2) Model 1 computed odds ratios without any statistical adjustment in unmatched participants. Model 2 computed odds ratios adjusting for covariates using multivariate logistic regressions in unmatched participants. Model 3 computed coefficient adjusting for covariates using fixed effect model in unmatched participants. Model 4 computed odds ratios without any statistical adjustments in matched participants. Model 5 computed odds ratios adjusting for covariates in multivariate logistic regressions in matched participants. Model 6 computed coefficient adjusting for covariates using fixed effect model in matched participants.

3) 1:2 matching was used to match male veterans with individuals without military experience.

**Table S7.** The association between the military experience and depression in male veterans before and after propensity score matching in junior college and above and below junior college group.

| **Analysis** | **Models** | **Junior college and above** | | | **Below junior college** | | |
| --- | --- | --- | --- | --- | --- | --- | --- |
|  |  | **OR/Coef. (95%, CI)** | **P-value** | **N** | **OR/Coef.**  **(95% CI)** | **P-value** | **N** |
| **Before**  **matching** | **Model 1**  **Crude** | 0.56  (0.34~0.92) | 0.023 | 1542 | 0.66  (0.56~0.79) | <0.001 | 11226 |
|  | **Model 2**  **Logistic** | 0.71  (0.42~1.20) | 0.201 | 1542 | 0.77  (0.64~0.91) | 0.003 | 11226 |
|  | **Model 3**  **Fixed effect** | -0.07  (-0.19~0.04) | 0.200 | 1542 | -0.06  (-0.10~-0.02) | 0.002 | 11226 |
| **After**  **matching** | **Model 4**  **Crude** | 0.49  (0.25~0.94) | 0.032 | 173 | 0.67  (0.53~0.85) | 0.001 | 1290 |
|  | **Model 5**  **Logistic** | 0.46  (0.23~0.92) | 0.027 | 173 | 0.69  (0.54~0.88) | 0.003 | 1290 |
|  | **Model 6**  **Fixed effect** | -0.15  (-0.29~-0.003) | 0.045 | 173 | -0.07  (-0.12~-0.01) | 0.012 | 1290 |

Notes:

1) *p<0.05, **p<0.01, ***p<0.001. Coef., coefficient; OR, odds ratio.

2) Model 1 computed odds ratios without any statistical adjustment in unmatched participants. Model 2 computed odds ratios adjusting for covariates using multivariate logistic regressions in unmatched participants. Model 3 computed coefficient adjusting for covariates using fixed effect model in unmatched participants. Model 4 computed odds ratios without any statistical adjustments in matched participants. Model 5 computed odds ratios adjusting for covariates in multivariate logistic regressions in matched participants. Model 6 computed coefficient adjusting for covariates using fixed effect model in matched participants.

3) 1:2 matching was used to match male veterans with individuals without military experience.

**Table S8.** The association between the military experience and depression in male veterans before and after propensity score matching in rural and urban group.

| **Analysis** | **Models** | **Rural** | | | **Urban** | | |
| --- | --- | --- | --- | --- | --- | --- | --- |
|  |  | **OR/Coef. (95%, CI)** | **P-value** | **N** | **OR/Coef.**  **(95% CI)** | **P-value** | **N** |
| **Before**  **matching** | **Model 1**  **Crude** | 0.74  (0.57~0.96) | 0.026 | 6455 | 0.63  (0.51~0.77) | <0.001 | 6313 |
|  | **Model 2**  **Logistic** | 0.83  (0.63~1.09) | 0.178 | 6455 | 0.77  (0.62~0.96) | 0.018 | 6313 |
|  | **Model 3**  **Fixed effect** | -0.04  (-0.09~0.01) | 0.158 | 6455 | -0.06  (-0.11~-0.01) | 0.010 | 6313 |
| **After**  **matching** | **Model 4**  **Crude** | 0.85  (0.61~1.19) | 0.349 | 710 | 0.68  (0.52~0.89) | 0.005 | 969 |
|  | **Model 5**  **Logistic** | 0.83  (0.59~1.17) | 0.290 | 710 | 0.68  (0.51~0.89) | 0.005 | 969 |
|  | **Model 6**  **Fixed effect** | -0.03  (-0.10~0.04) | 0.342 | 710 | -0.08  (-0.15~-0.02) | 0.006 | 969 |

Notes:

1) *p<0.05, **p<0.01, ***p<0.001. Coef., coefficient; OR, odds ratio.

2) Model 1 computed odds ratios without any statistical adjustment in unmatched participants. Model 2 computed odds ratios adjusting for covariates using multivariate logistic regressions in unmatched participants. Model 3 computed coefficient adjusting for covariates using fixed effect model in unmatched participants. Model 4 computed odds ratios without any statistical adjustments in matched participants. Model 5 computed odds ratios adjusting for covariates in multivariate logistic regressions in matched participants. Model 6 computed coefficient adjusting for covariates using fixed effect model in matched participants.

3) 1:2 matching was used to match male veterans with individuals without military experience.

**Table S9.** The association between the military experience and individual depression score in male veterans before and after propensity score matching in unmarried and married group.

| **Analysis** | **Models** | **Unmarried** | | | **Married** | | |
| --- | --- | --- | --- | --- | --- | --- | --- |
|  |  | **Coef.**  **(95%, CI)** | **P-value** | **N** | **Coef.**  **(95%, CI)** | **P-value** | **N** |
| **Before**  **matching** | **Model 1**  **Crude** | 0.69  (-1.19~2.58) | 0.470 | 2204 | -1.54  (-2.16~-0.92) | <0.001 | 10564 |
|  | **Model 2**  **Logistic** | 0.63  (-1.22~2.48) | 0.503 | 2204 | -1.04  (-1.66~-0.42) | 0.001 | 10564 |
|  | **Model 3**  **Fixed effect** | 0.64  （-1.21~2.50） | 0.495 | 2204 | -1.04  (-1.66~-0.42) | 0.001 | 10564 |
| **After**  **matching** | **Model 4**  **Crude** | 0.50  （-2.03~3.02） | 0.700 | 2204 | -1.58  (-2.36~-0.79) | <0.001 | 10564 |
|  | **Model 5**  **Logistic** | 0.67  （-1.92~3.25） | 0.612 | 2204 | -1.51  (-2.27~-0.75) | <0.001 | 10564 |
|  | **Model 6**  **Fixed effect** | 0.73  （-1.92~3.37） | 0.589 | 2204 | -1.48  (-2.24~-0.71) | <0.001 | 10564 |

Notes:

1) *p<0.05, **p<0.01, ***p<0.001. Coef., coefficient.

2) Model 1 computed coefficient without any statistical adjustment in unmatched participants. Model 2 computed coefficient adjusting for covariates using multivariate logistic regressions in unmatched participants. Model 3 computed coefficient adjusting for covariates using fixed effect model in unmatched participants. Model 4 computed coefficient without any statistical adjustments in matched participants. Model 5 computed coefficient adjusting for covariates in multivariate logistic regressions in matched participants. Model 6 computed coefficient adjusting for covariates using fixed effect model in matched participants.

3) 1:2 matching was used to match male veterans with individuals without military experience.

**Table S10.** The association between the military experience and individual depression score in male veterans before and after propensity score matching in junior college and above and below junior college group.

| **Analysis** | **Models** | **Junior college and above** | | | **Below junior college** | | |
| --- | --- | --- | --- | --- | --- | --- | --- |
|  |  | **Coef.**  **(95%, CI)** | **P-value** | **N** | **Coef.**  **(95%, CI)** | **P-value** | **N** |
| **Before**  **matching** | **Model 1**  **Crude** | -1.87  (-3.45~-0.30) | 0.020 | 1542 | -1.38  （-2.02~-0.73） | <0.001 | 11226 |
|  | **Model 2**  **Logistic** | -1.10  (-2.72~0.52) | 0.185 | 1542 | -1.12  (-1.76~-0.48) | 0.001 | 11226 |
|  | **Model 3**  **Fixed effect** | -1.02  (-2.65~0.60) | 0.215 | 1542 | -1.13  (-1.77~-0.49) | 0.001 | 11226 |
| **After**  **matching** | **Model 4**  **Crude** | -2.17  (-4.18~-0.17) | 0.034 | 173 | -1.49  (-2.35~-0.62) | 0.001 | 1290 |
|  | **Model 5**  **Logistic** | -1.86  (-3.81~0.08) | 0.060 | 173 | -1.28  (-2.13~-0.43) | 0.003 | 1290 |
|  | **Model 6**  **Fixed effect** | -1.73  (-3.70~0.25) | 0.086 | 173 | -1.18  (-2.04~-0.33) | 0.007 | 1290 |

Notes:

1) *p<0.05, **p<0.01, ***p<0.001. Coef., coefficient.

2) Model 1 computed coefficient without any statistical adjustment in unmatched participants. Model 2 computed coefficient adjusting for covariates using multivariate logistic regressions in unmatched participants. Model 3 computed coefficient adjusting for covariates using fixed effect model in unmatched participants. Model 4 computed coefficient without any statistical adjustments in matched participants. Model 5 computed coefficient adjusting for covariates in multivariate logistic regressions in matched participants. Model 6 computed coefficient adjusting for covariates using fixed effect model in matched participants.

3) 1:2 matching was used to match male veterans with individuals without military experience.

**Table S11.** The association between the military experience and individual depression score in male veterans before and after propensity score matching in rural and urban group.

| **Analysis** | **Models** | **Rural** | | | **Urban** | | |
| --- | --- | --- | --- | --- | --- | --- | --- |
|  |  | **Coef.**  **(95%, CI)** | **P-value** | **N** | **Coef.**  **(95%, CI)** | **P-value** | **N** |
| **Before**  **matching** | **Model 1**  **Crude** | -0.85  (-1.82~0.12) | 0.086 | 6455 | -1.53  (-2.27~-0.78) | <0.001 | 6313 |
|  | **Model 2**  **Logistic** | -0.72  (-1.68~0.24) | 0.142 | 6455 | -0.95  (-1.70~-0.21) | 0.012 | 6313 |
|  | **Model 3**  **Fixed effect** | -0.72  (-1.68~0.24) | 0.141 | 6455 | -0.96  (-1.71~-0.22) | 0.011 | 6313 |
| **After**  **matching** | **Model 4**  **Crude** | -0.93  (-2.19~0.33) | 0.149 | 710 | -1.77  (-2.75~-0.79) | <0.001 | 969 |
|  | **Model 5**  **Logistic** | -1.02  (-2.24~0.20) | 0.100 | 710 | -1.68  (-2.62~-0.73) | 0.001 | 969 |
|  | **Model 6**  **Fixed effect** | -1.01  (-2.24~0.21) | 0.105 | 710 | -1.70  (-2.65~-0.76) | <0.001 | 969 |

Notes:

1) *p<0.05, **p<0.01, ***p<0.001. Coef., coefficient.

2) Model 1 computed coefficient without any statistical adjustment in unmatched participants. Model 2 computed coefficient adjusting for covariates using multivariate logistic regressions in unmatched participants. Model 3 computed coefficient adjusting for covariates using fixed effect model in unmatched participants. Model 4 computed coefficient without any statistical adjustments in matched participants. Model 5 computed coefficient adjusting for covariates in multivariate logistic regressions in matched participants. Model 6 computed coefficient adjusting for covariates using fixed effect model in matched participants.

3) 1:2 matching was used to match male veterans with individuals without military experience.

**Figure S1** Standardized differences between the veteran and non-veteran groups before and after the 1:2 nearest-neighbor matching with replacement in different subgroups.


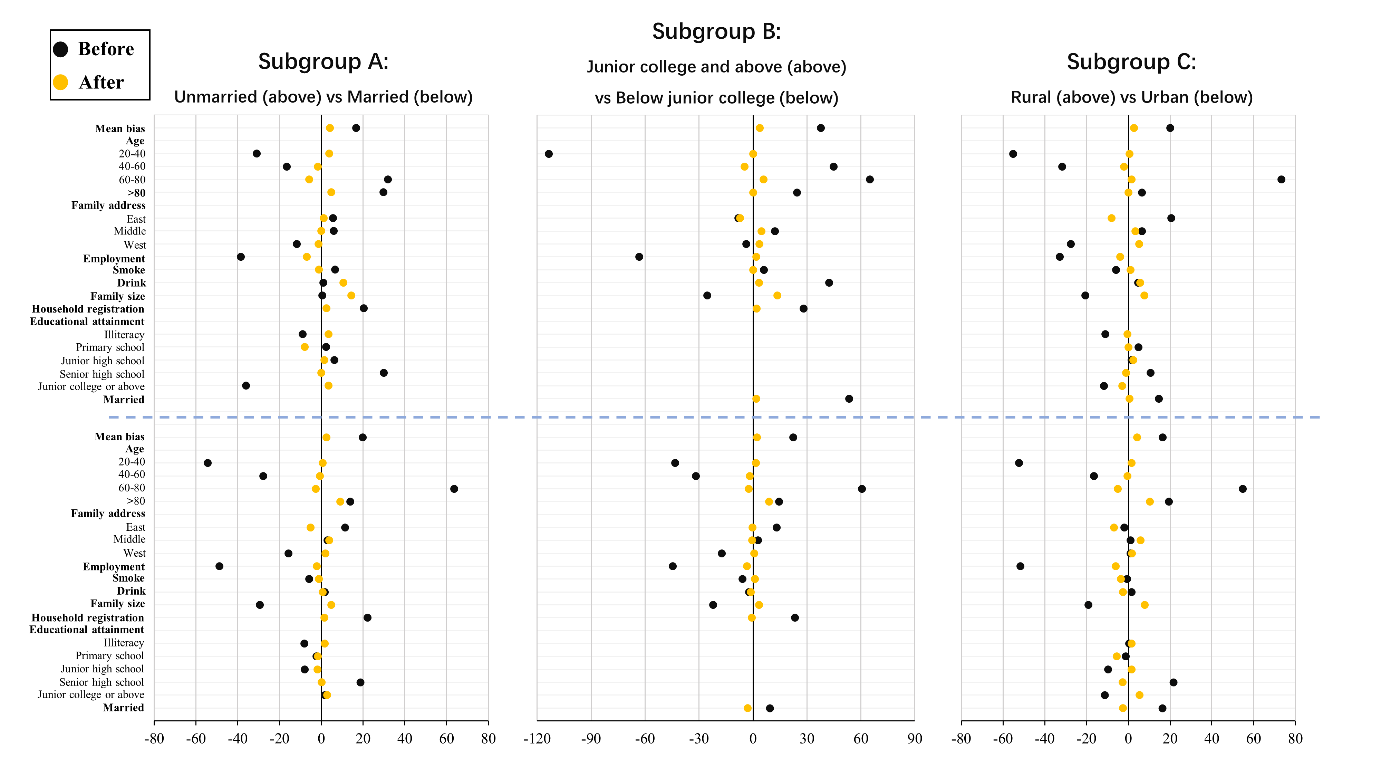


Notes:

1) The black dots and yellow dots represent standardized differences before and after matching respectively.

2) Details are provided in Supplementary Table S5.
